# Comparative analysis of the *Saccharomyces cerevisiae *and *Caenorhabditis elegans *protein interaction networks

**DOI:** 10.1186/1471-2148-5-23

**Published:** 2005-03-18

**Authors:** Ino Agrafioti, Jonathan Swire, James Abbott, Derek Huntley, Sarah Butcher, Michael PH Stumpf

**Affiliations:** 1 Theoretical Genomics Group, Centre for Bioinformatics, Division of Molecular Biosciences, Imperial College London, Wolfson Building, SW7 2AZ, London, UK; 2 Bioinformatics Support Service, Centre for Bioinformatics, Division of Molecular Biosciences, Imperial College London, Wolfson Building, SW7 2 AZ, London, UK

## Abstract

**Background:**

Protein interaction networks aim to summarize the complex interplay of proteins in an organism. Early studies suggested that the position of a protein in the network determines its evolutionary rate but there has been considerable disagreement as to what extent other factors, such as protein abundance, modify this reported dependence.

**Results:**

We compare the genomes of *Saccharomyces cerevisiae *and *Caenorhabditis elegans *with those of closely related species to elucidate the recent evolutionary history of their respective protein interaction networks. Interaction and expression data are studied in the light of a detailed phylogenetic analysis. The underlying network structure is incorporated explicitly into the statistical analysis. The increased phylogenetic resolution, paired with high-quality interaction data, allows us to resolve the way in which protein interaction network structure and abundance of proteins affect the evolutionary rate. We find that expression levels are better predictors of the evolutionary rate than a protein's connectivity. Detailed analysis of the two organisms also shows that the evolutionary rates of interacting proteins are not sufficiently similar to be mutually predictive.

**Conclusion:**

It appears that meaningful inferences about the evolution of protein interaction networks require comparative analysis of reasonably closely related species. The signature of protein evolution is shaped by a protein's abundance in the organism and its function and the biological process it is involved in. Its position in the interaction networks and its connectivity may modulate this but they appear to have only minor influence on a protein's evolutionary rate.

## Background

Studies of the evolutionary history of protein interaction network (PIN) data have produced an almost bewildering range of (partially) contradictory results [1-6,8-12]. While PIN data is notoriously prone to false positive and negative results [5,13], reasons for disagreements are probably more diverse than just the quality of the interaction data. Failure to account for protein abundance – as measured by gene expression levels, or by proxy, the codon-adaptation index – has been criticized [3]; the choice of species for comparative analysis will also affect any evolutionary inferences as shown by Hahn *et al. *[12]. This may either be due to loss of power (*e.g. *fewer reliably identified orthologues between more distantly related species) or to differences in underlying PINs in distantly related species. Below, for example, we will show that results obtained from a comparison between the two hemiascomycetes *Saccharomyces cerevisiae *and *Candida albicans *differ considerably from those obtained using a distant *S. cerevisiae *– *Caenorhabditis elegans *comparison. Finally, it has recently been shown that many studies may have suffered from the fact that present network data, and this is in particular true for PINs, are random samples from much larger networks. Unless these subnets are adequate representations of the overall network, their structural properties (such as node connectivity) may differ quite substantially from that of the nodes in the global network. This is, for example, the case for so-called scale-free network models [14].

Moreover, many studies have ignored the underlying network structure [15] in the statistical analysis. The network, however, introduces dependencies between connected proteins which should not be ignored. Fraser *et al. *[2] for example find that (i) there is a negative correlation between a protein's evolutionary rate and its connectivity *k *(the number of its interactions), (ii) connected proteins have positively correlated evolutionary rates, and (iii) connected proteins do not have correlated connectivities. All three statements cannot, of course, be strongly true simultaneously. Here we observe only relatively weak – though statistically significant – correlations between connectivity and evolutionary rate. We will argue that in a regression framework [16] some of these quantities contain very little information indeed about the corresponding properties of their interaction partners. Furthermore, we will demonstrate that when analyzing network data the network structure must be included into the analysis from the outset. Here we will first perform an evolutionary analysis of the yeast and nematode PIN data available in the DIP database [7], a hand-curated dataset combining information from a wide range of sources, followed by a comparison of the two datasets. When making comparisons between yeast species and between nematode species, we use only a single PIN dataset – for *S. cerevisiae *and *C. elegans*, respectively – and take comfort from the observation of Hahn *et al. *[12] who find that evolutionary analysis involving closely related reference taxa produces consistent results. Previously, topological comparisons of biological network data from different species have been made [17] but here we focus on shared evolutionary characteristics of PINs in the two species. We would expect at least some level of similarity of biological networks between species; but the more distantly related two organisms are, the more changes can have accumulated in their respective molecular networks. Thus, the depth of the phylogeny can affect the evolutionary analysis of PINs; it is, for example, unlikely that PINs have been conserved over large evolutionary time-scales.

## Results

### Evolutionary analysis of the *S. cerevisiae *PIN

**Figure 1:**
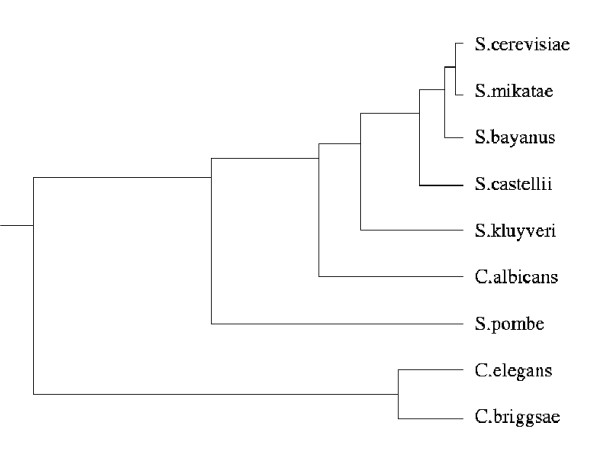
**Phylogeny of the organisms used in the study**. The evolutionary relationship of the organisms used in this study. The last common ancestor of the ascomycetes in this phylogeny has been estimated to have lived approximately 330 million years ago. For the nematodes only two annotated genomes were available: their last common ancestor is believed to have lived approximately 100 million years ago.

For the evolutionary analysis of the yeast PIN we use a panel of related yeast species: *Saccharomyces mikatae*, *Saccharomyces bayanus*, *Saccharomyces casteliii*, *Saccharomyces kluyveri*, *C. albicans *and *Schizosaccharomyces pombe *(see Methods section); the evolutionary relationship between these species is shown in figure 1. We thus focus on relatively recent evolutionary change which allows us to study the effects of the network structure on the rate of evolution more directly than *e.g. *distant comparisons of *S. cerevisiae *and *C. elegans*, which may, after all, have different PINs.

### Connectivity, expression and evolutionary rates in the *S. cerevisiae *PIN

For most protein sequences we have not been able to identify orthologues in all yeast species used in this analysis. We therefore defined the averaged relative evolutionary rate *R *(see Eqn. (1) in the *Methods *section) which allows us to make comparisons for 4124 out of the 4773 yeast genes for which we have interaction data.

**Figure 2:**
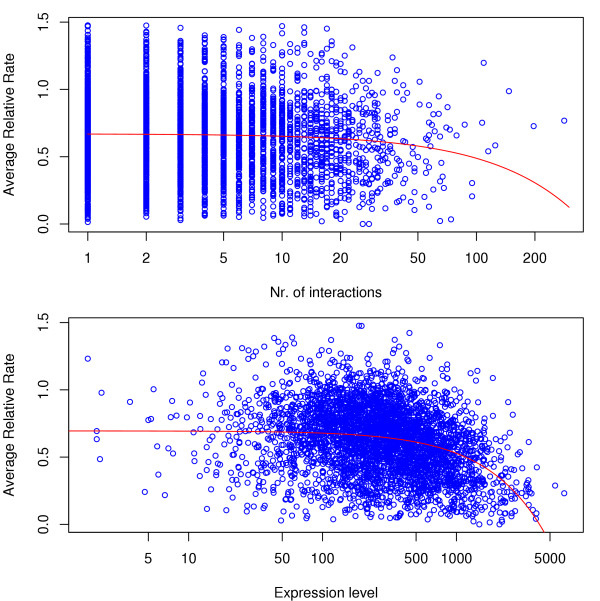
**Dependence of evolutionary rate in ascomycetes on the number of protein interactions and expression level**. The averaged relative rate *R *decreases with increasing number of interaction partners (*τ *≈ -0.06) and the expression level (*τ *≈ -0.23). The 95% bootstrap intervals for Kendall's *τ *values obtained from the six species comparisons are always negative (see table 1). The linear regression curves (red) appear concave on the log-transformed *x*-axis.

**Figure 3:**
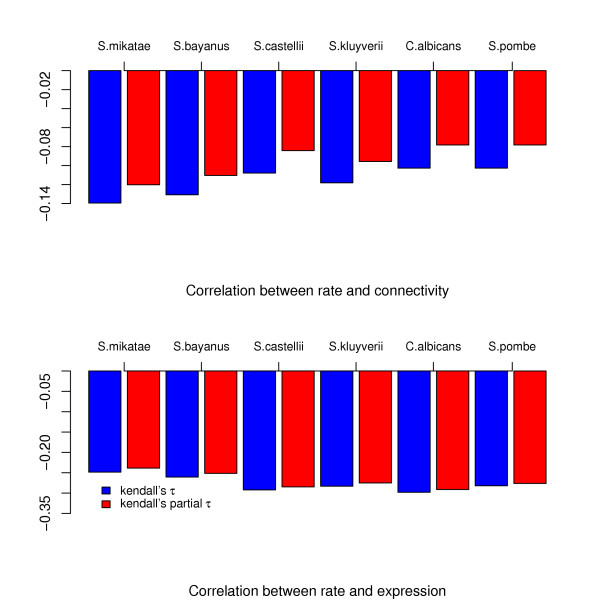
**Correlation and partial correlation between evolutionary rate, number of interactions and expression level**. Kendall's rank correlation (blue) and partial rank correlation coefficients (red) between *R *and the number of interactions (correcting the partial *τ *for expression level) and expression (correcting for the number of interactions).

In figure 2 we show the dependence between inferred evolutionary rates and connectivities and expression levels, respectively. Our comparative analysis found statistically significant, though small, negative correlation, measured by Kendall's *τ*, between estimated evolutionary rates and a protein's number of interactions. In table 1 and figure 3 we observe that comparisons with all species support this notion We furthermore estimated approximate confidence intervals for *τ *from 1000 bootstrap replicates [18] (shown in table 1).

**Table 1:** Evolutionary analysis of *S. cerevisiae *Correlations between evolutionary rate, number of connections and expression level of proteins and the confidence intervals for Kendall's *τ *statistic obtained for the different ascomycete species. Values of *τ *that have associated p-values < 0.01 are highlighted in bold. *X*1 denotes correlation with evolutionary rate obtained from a pairwise sequence comparison between *S. cerevisiae *and species *X*; *X*2 differs from *X*1 only in that the evolutionary rate was obtained using a maximum likelihood estimate. *M *denotes a rate obtained with respect to *S. mikatae*, *B *to *S. bayanus*, *C *to *S. castellii*, *K *to *S. kluyveri*, *A *to *C. albicans*, and *P *to *S. pombe*.

	Species comparison											
	M1	M2	B1	B2	C1	C2	K1	K2	A1	A2	P1	P2

Connectivity	**-0.13**	**-0.16**	**-0.13**	**-0.14**	**-0.11**	**-0.12**	**-0.12**	**-0.14**	**-0.08**	**-0.08**	**-0.10**	**-0.10**
2.5-%	-0.17	-0.19	-0.16	-0.17	-0.13	-0.14	-0.15	-0.17	-0.11	-0.11	-0.14	-0.13
97.5-%	-0.11	-0.13	-0.11	-0.12	-0.08	-0.09	-0.08	-0.10	-0.06	-0.05	-0.07	-0.07

Expression	**-0.25**	**-0.30**	**-0.26**	**-0.29**	**-0.29**	**-0.32**	**-0.28**	**-0.32**	**-0.28**	**-0.28**	**-0.30**	**-0.28**
2.5-%	-0 28	-0 33	-0 28	-0 30	-0 31	-0 34	-0 32	-0 35	-0 31	-0 30	-0 33	-0 31
97.5-%	-0.22	-0.27	-0.24	-0.27	-0.27	-0.30	-0.25	-0.29	-0.26	-0.25	-0.27	-0.25

Observed negative correlations between estimated evolutionary rates and the expression level – which have been reported previously by Pal *et al. *[19] – are more pronounced. Equally, *k*, the number of a protein's interactions, and expression levels are also correlated (*τ *= 0.09). There has been considerable controversy as to whether the effect of a protein's connectivity can be studied independently of expression levels (see *e.g. *[3,4]). The observed values of *τ *suggest that expression levels are better predictors of the evolutionary rate than are connectivities. Calculating partial rank correlation coefficients, *τ*_*p*_, provides further evidence for this: correcting for expression reduces the correlation between the evolutionary rate *R *(or any of the individual rates) and the number of interactions, as is apparent from figure 3. As the phylogenetic distance between species increases, the negative partial correlation between evolutionary rate and connectivity decreases compared to the uncorrected rank correlation measure *τ*.

In the supplementary tables S1-S3 [see Additional file 1] we show the evolutionary rates for the different functional categories, processes and cellular compartments (taken from *Gene Ontology *(GO) [20]. Interestingly, once the effects of expression and protein function on the estimated evolutionary rate are taken into account the dependence of the latter on connectivity in a generalized linear regression model [16] (where we log-transformed the expression level to obtain an approximately normal distribution) is considerably reduced. This can be assessed formally using the Akaike information criterion (AIC) [21] on the sub-models where one of the terms has been dropped (see methods). For the full model we obtain AIC = -407.4. Dropping expression from the model results in AIC = -196.9, indicating that a substantial amount of information about the evolutionary rate is contained in the expression levels. Dropping the other terms individually while retaining the rest results in: AIC = -392.9 if the connectivity is dropped from the statistical model, and AIC = -352.6 (process), -250.1 (function) and -392.7 (compartment). We thus have the following order of statistically inferred impact on the evolutionary rate (with a slight abuse of the notation): expression>function>process>connectivity≈compartment. Using the rates obtained from comparisons with the individual species results in the same ordering.

### Evolution of interacting proteins in *S. cerevisiae*

So far we have treated nodes/proteins as independent (using only their connectivities in the analysis) but we will now consider the extent to which interactions introduce dependencies into the data. It is intuitively plausible that interacting proteins have similar evolutionary rates, and this has indeed been reported by Fraser *et al. *[2,22] and studied by others, too, *e.g. *[3,12]. Just like them we find that evolutionary rate decreases with connectivity; we also observe that the connectivities of interacting proteins are anti-correlated in yeast (*τ *≈ -0.03 with *p *< 10^-8^). This is well explained from the statistical theory of networks [14,23], as well as structural analyses of PIN data, where it is found that highly connected proteins form hubs which connect sparsely connected proteins.

**Figure 4:**
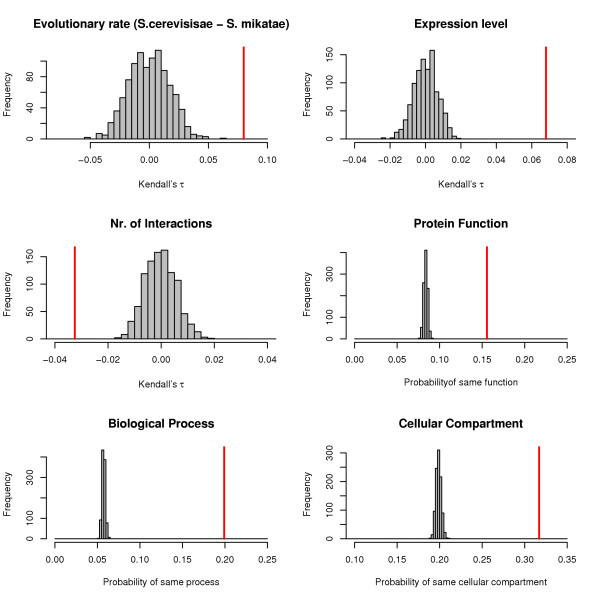
**Statistical dependencies of interacting proteins in *S. cerevisiae***. Bootstrap distributions of Kendall's *τ *between evolutionary rates, expression levels and numbers of interactions and probabilities that protein function and the processes and cellular compartments by which proteins are classified are identical for a pair of interacting proteins. The grey histograms show the distribution of the statistics obtained from 1000 bootstrap replicates and the red vertical lines indicate the observed value. The bootstrap procedure was constrained such that each sample reproduced the degree distribution of the observed PIN.

Taken together this would mean that the evolutionary rates of connected proteins should also be anti-correlated. This is, however, not the case when we look at the yeast PIN, where we find that evolutionary rates of interacting proteins are positively correlated as measured by Kendall's *τ*. The correlations we observe are only relatively weak (even though they are significant) *τ *≈ 0.05 – 0.10 with *p *< 10^-8^. In figure 4 we show the distribution of the *τ *rank correlation under the correct network Null model (see methods) for rates, expression levels and connectivities of interacting proteins. The observed value always lies outside the distribution of the expected values. Also shown in the figure are the probabilities that two interacting proteins have identical GO-classifications for function, process and cellular compartment, respectively. Again the observed probabilities lie outside the distribution under the Null model.

Correlation, even partial correlation, may, however, be an inadequate statistical measure if the data is structured (as in a network); one should then rather focus on the power of a factor such as expression level or connectivity to predict evolutionary rates. We assess this formally through the use of statistical regression models which describe the evolutionary rate of one protein as a function of the rate of its interacting partner, as well as of its expression level, number of interactions, function, process and cell compartment. The AIC, which for the full model is AIC = -2397.6, allows us to order the factors by the information they contain about a protein's evolutionary rate. The order (and the respective AIC value on dropping the factor from the model) is as follows: Expression (AIC = -1399.6), function (AIC = -1445.9), process (AIC = -1956.6), cellular compartment (AIC = -2226.6), connectivity (AIC = -2316.8), and the rate of one of its interaction partners (AIC = -2397.0). Note that, measured by the AIC, the evolutionary rate of an interaction partner provides virtually no additional information about a protein's own evolutionary rate, once the protein's own expression level, function and process have been taken into account.

Thus, in summary, we observe that the evolutionary rate of yeast proteins is inversely related both to their connectivity in the PIN and to their expression levels, with expression levels having a greater impact on a protein's evolutionary rate than connectivities. Finally, while there is statistically significant correlation between the rates of interacting proteins, the rate of one interaction partner carries very little information about the rate of the other protein if other factors are taken into account.

### Evolutionary analysis of the *C. elegans *PIN

In the evolutionary analysis of *C. elegans *we use *C. briggsae*, the only other congeneric nematode for which high quality whole-genome data is available. Since nematodes are multicellular, care has to be taken when analysing the effects of gene expression on evolutionary rate, as expression levels will vary considerably between tissues and, indeed, between different stages of the nematode life cycle. Because codon usage bias as a selective response increasing translational efficiency should be driven by the overall expression level of a protein integrated over both tissue and time, the codon-adaptation index (CAI; see Methods and [24]) can serve as a meaningful averaged quantity reflecting overall integrated expression levels better than a direct measurement of mRNA expression level data obtained from any single tissue type.

### Connectivity, expression and evolutionary rates in the *C. elegans *PIN

**Figure 5:**
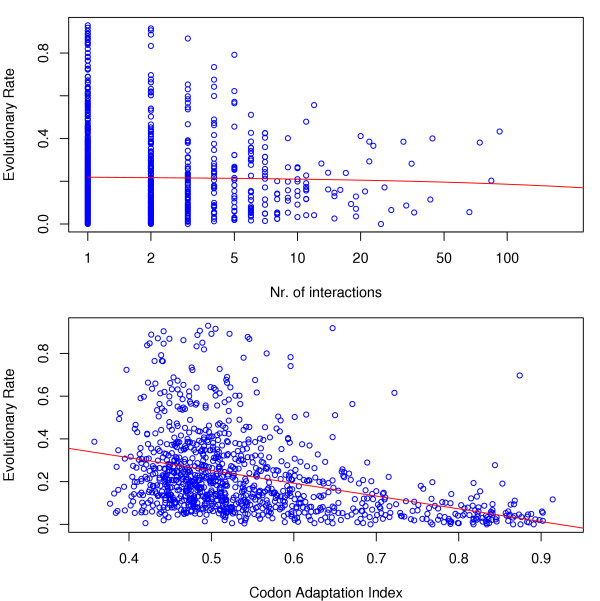
**Dependence of evolutionary rate in nematodes on the number of protein interactions and CAI**. The estimated evolutionary rate decreases with increasing number of interaction partners (*τ *≈ -0.03) and the expression level (*τ *≈ -0.30). We have again transformed the *x*-axis in the scatterplot of rate vs. connectivity which leads to the concave shape of the regression line (red).

The correlation of evolutionary rate and connectivity is somewhat reduced compared to *S. cerevisiae *with a point estimate of *τ *= -0.05 with a 95% bootstrap CI of [-0.097, -0.017]. Anti-correlation between the CAI measure of expression and evolutionary rates is again much more pronounced with *τ *≈ -0.30 and approximate bootstrap CIs of [-0.333, -0.264]. The resulting scatter plots of rate vs. connectivity and rate vs. CAI are shown in figure 5.

Partial correlation coefficients again show that the influence of expression is greater than that of connectivity: *τ*_*p *_≈ -0.03 for the partial correlation measure between rate and connectivity, while *τ*_*p *_≈ -0.30 if the correlation between expression (CAI) and rate is corrected for connectivity. This is confirmed by performing an ANOVA [25] on the regression between rate, CAI and connectivity where no significant correlation can be found between rate and connectivity (*p *≈ 0.62). Generalized linear regression modelling shows that measured by the AIC a model in which the rate depends only on the CAI but not on the connectivity (AIC = -660.5) is more powerful than a model in which the rate depends on both connectivity and CAI (AIC = -618.4). In the absence of extensive GO data we find that the CAI is the only statistically significant predictor for a protein's evolutionary rate.

### Evolution of interacting proteins in *C. elegans*

Comparing properties of interacting proteins we again find a negative correlation between their respective connectivities (*τ *= -0.07) and a weaker positive correlation between their evolutionary rates (*τ *= 0.03).

**Figure 6:**
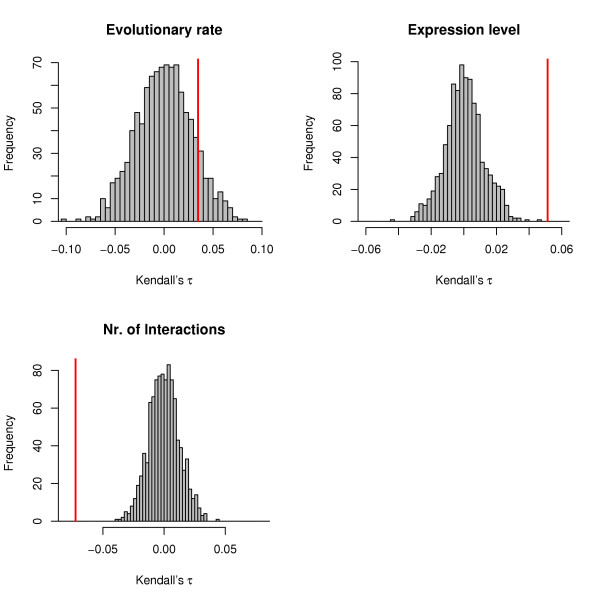
**Statistical dependencies of interacting proteins in *C. elegans***. Bootstrap distributions of Kendall's *τ *between evolutionary rates, expression levels and numbers of interactions. The grey histograms show the distribution of *τ *obtained in 1000 bootstrap replicates and the red lines indicate the observed value. The bootstrap procedure was constrained such that each sample reproduced the degree distribution of the observed PIN.

The corresponding 95% bootstrap CI for *τ *does, however, include 0 and negative values; thus there is no statistical basis for concluding that evolutionary rates of interacting proteins are correlated in *C. elegans *even if we consider only the rank correlation measure. In figure 6 the distribution of *τ *under the correct Null model (see methods) confirms this result as the observed correlation between the evolutionary rates of interacting proteins falls into the 95% confidence interval obtained from the Null model. Expression levels are, however, significantly correlated and connectivities remain significantly anti-correlated. Regression models, equivalent to those performed for yeast, confirm the negligible information a protein's evolutionary rate contains about the evolutionary rate of an interacting protein.

In summary, for *C. elegans *we find that expression, even if measured indirectly through the CAI, is a better predictor about a protein's evolutionary rate than connectivity and GO classifications. The evolutionary rates of connected proteins do not appear to be correlated.

### Comparing the PINs of *S. cerevisiae *and *C. elegans*

It is instructive to compare the PINs of the two model organisms, yeast and worm, directly. We have therefore used our earlier approach of identifying and analysing orthologues to the yeast and nematode PIN data. While we are, of course, aware that this may be problematic given the two or three billion years of evolutionary history separating the two organisms, it should serve as a useful illustration of the amount of information one model-organism is likely to provide about another (including, of course, humans). Using this approach we found a total of 524 pairs of orthologues. These we aligned and from the alignments we estimated evolutionary rates. For all of these proteins we have PIN data and for most we also have information about their expression levels in the two species. The results are summarized in tables 2 and 3. Although they essentially agree with the earlier results, they do suggest that the choice of species used for inferring the evolutionary rate can influence the analysis. For example, the partial correlation between interaction and evolutionary rate (calculated directly from the *S. cerevisiae *– *C. elegans *amino acid sequence comparison) accounting for expression is much less reduced compared with the simple correlation coefficient (*τ*_*p *_= -0.20 in *S. cerevisiae*, and *τ*_*p *_= -0.10 in *C. elegans*) than when evolutionary rates are calculated using more closely related target species. Over long evolutionary distances it appears as if connectivity and expression level act almost independently. However, the more reliable comparisons of the previous section suggest that this is not the case.

**Table 2:** Correlations obtained from a direct comparison of *S. cerevisiae *with *C. elegans *Orthologues in the *S. cerevisiae *and *C. elegans *PINs where identified by reciprocal BLAST searches and evolutionary rates, estimated previously (see table 1), were analysed for correlation between evolutionary rate, the number of interactions and expression levels. We also performed an analysis with evolutionary rates estimated directly from the distant *S. cerevisiae *and *C. elegans *comparison.

**Comparison**	**Evolutionary Rate obtained from closely related species**		**Evolutionary Rate obtained from *S. cerevisiae*- *C. elegans***	
	
	*S. cerevisiae*	*C. elegans*	*S. cerevisiae*	*C. elegans*
Nr. of Interactions	**-0.11**	**-0.13**	**-0.20**	**-0.10**
2.5-percentile	-0.20	-0.23	-0.26	-0.19
97.5-percentile	-0.02	-0.04	-0.14	-0.03

Expression	**-0.33**	**-0.44**	**-0.25**	**-0.42**
2.5-percentile	-0.41	-0.50	-0.32	-0.47
97.5-percentile	-0.24	-0.36	-0.19	-0.37

**Table 3:** Correlations between orthologous proteins in the *S. cerevisiae *and *C. elegans *PINs Observed rank correlations (measured by Kendall's *τ*) for evolutionary rates (measured with respect to *S.mikatae *and *C. briggsae*, respectively), connectivity and protein expression level (estimated by mRNA expression level in *S. cerevisiae *and CAI in *C. elegans*).

**Quantity**	**Observed ***τ*	**95% CI**
Evolutionary Rate	0.24	0.12–0.35
Connectivity	0.07	0.001–0.14
Expression	0.32	0.26–0.39

Comparing properties of orthologous proteins we find that their expression levels (using the CAI as a proxy in *C. elegans*) show the strongest correlation while their respective PIN connectivities show the lowest value for Kendall's *τ *statistic. This may be due to the noise in the PIN data or the incomplete nature of present PIN data sets. We expect that the relatively small proportion of *C. elegans *proteins included in the DIP database will also lead to an inaccurate representation of the *C. elegans *PIN.

## Discussion

There are considerable differences between the various published studies [2-4,12], both in terms of protein interaction data and phylogenetic comparisons. We therefore focus on closely related species for both *S. cerevisiae *and *C. elegans *in the evolutionary analysis, since we probably have to assume that the underlying PIN is relatively more conserved over short evolutionary distances. While we found some evidence that highly connected proteins evolve more slowly than sparsely connected proteins, (i) the negative correlation between rate and expression level is more pronounced, (ii) in *S. cerevisiae *and *C. elegans *connectivity turns out to be a worse statistical predictor of the evolutionary rate than expression. For *S. cerevisiae *we also find that protein function and the principal biological process a protein is involved in have a greater impact on the evolutionary rate of a protein than its connectivity.

We believe that the importance of expression over connectivity in determining the evolutionary rate may be due to three factors. First, highly abundant genes are perhaps more visible to purifying selection [26], which might tend to lower the rate at which they evolve. Second, and more importantly, highly expressed genes, which are under selection for translational efficiency, use only a small subset of the cognate codons for a particular amino acid (this, incidentally, is exploited in the construction of the CAI), and because this subset is often the same even in phylogenetically remote organisms – for example, for those amino acids encoded by nnU and nnC (*e.g. *phenylalanine or cysteine), nnC is almost universally preferred – the silent substitution rate is reduced. Third, the replacement substitution rate in highly expressed proteins should also be reduced for a similar reason to selection for translational efficiency at silent sites: selection for more cheaply synthesised amino acids at replacement sites [27]. This can be shown to lead to the avoidance of amino acids which are metabolically expensive to synthesise at functionally-unconstrained sites in highly expressed proteins, which reduces the set of acceptable amino acids at such sites and thereby lowers the replacement substitution rate compared with that at functionally-unconstrained sites in low expression proteins [28].

We have also applied an improved resampling procedure to the analysis of correlation between rates and expression levels of connected proteins. In our analysis we treated properties of the network as a confounding variable and in addition to studying correlations we also show how informative properties of one protein are about properties of its interaction partners. We find that the correct procedure broadens the resampling confidence intervals but that expression levels of interacting proteins remain considerably closer than would be expected by chance. Conversely we found no evidence of a correlation in the evolutionary rate of interacting proteins in *C. elegans *and only extremely weak evidence in *S. cerevisiae*. Our results also suggest, that the evolution of interacting proteins is not as tightly correlated as some researchers have proposed. This level of disagreement may be caused by uncertainties in the data or the fact that subnets sampled from larger networks inaccurately reflect the properties of the true network [29].

## Conclusion

We believe that the effects of the network structure on the evolution of proteins, and vice versa, is much more subtle than has previously been suggested. In the present dataset expression levels appear to have shaped a protein's evolutionary rate more than its connectivity. If we are happy to accept present PIN data with the necessary caution, then this observation is consistent with a scenario where expression levels are more conserved between species than are details of the interaction network. Nevertheless, we believe that it is important to consider the PIN and a protein's connectivity explicitly and from the outset in any statistical analysis as the underlying network appears to act as a confounding factor.

## Methods

### Data

#### Protein interaction data

The names and sequences for proteins with known interactions in Saccharomyces cerevisiae and Caenorhabditis elegans were retrieved from the Database of Interacting Proteins (DIP) on the 5th of July [7]. The database mainly contains information extracted from the research literature, but recently the database was enriched with information obtained by analysing structures of protein complexes deposited in PDB [30]. We have data for 4773 yeast proteins (comprising 15461 interactions) and 2386 nematode proteins (with 7221 interactions). While there have been recent attempts at quantifying levels of confidence in given protein interactions these generally lead to substantial decreases in sample size. For this reason we have therefore chosen to take the PIN data as it is deposited in the hand-curated DIP database (we have also performed analyses with such restricted subsets which agree with the results presented here).

#### Protein sequence data

In addition to *S. cerevisiae *sequences downloaded from the DIP, publicly available protein sequences of six other yeast species were investigated: *Saccharomyces pombe *[31], *Candida albicans *[32], *Saccharomyces mikatae, Saccharomyces bayanus, Saccharomyces kluyveri and Saccharomyces castellii *[33]. Genomic protein sequences for only one other Caenorhabditis species apart from *C. elegans *are publicly available, *C. briggsae *[34]. All sequence files were converted to searchable indexed databases; these are available from the authors.

#### Expression data

*S. cerevisiae *expression data came from Cho et al. [35] who characterised all mRNA transcript levels during the cell cycle of *S. cerevisiae. *mRNA levels were measured at 17 time points at 10 min intervals, covering nearly two full cell cycles [36]. The mean of these 17 numbers was taken to produce one general time-averaged expression level for each protein.

*C. elegans *is a multicellular organism in which different cells have different functions. This means that different proteins have different expression levels in different cells, which are present in different numbers, so taking a simple mean of the expression levels in a single cell type would be meaningless. In addition, *C. elegans *has a complex life-cycle, with different proteins being expressed in different stages of that cycle. Thus, an alternative way of calculating a single expression level for each protein had to be used. It has long been known that highly expressed genes tend to use only a limited number of codons thus displaying high codon bias. Sharp and Li [24] devised a measure for assessing the degree of deviation from a preferred pattern of usage estimated from the clustering of codon usage across proteins, which they called the Codon Adaptation Index (CAI). We adopt this measure as our expression level proxy.

### Methods

#### Phylogenetic analysis

The close relationship between the species considered here, apart from the distant comparison between *S. cerevisiae *and *C. elegans*, makes identification of orthologues relatively straightforward. Orthologous protein sequences were detected by reciprocal BLAST searches in the standard way. Multiple alignments of inferred orthologues were obtained using ClustalW.

Evolutionary rates were obtained using PAML (Phylogenetic Analysis by Maximum Likelihood) [37]. We used both the observed fraction of amino acid differences, referred to by *M*1, *B*1,... (where the letters refer to different species, see footnote to Table 1), and the distance related measure calculated from the trees inferred by PAML, referred to by *M*2, *B*2, .... Both rates are highly correlated *τ *≈ 0.9. In order to estimate the latter rate the phylogeny had to be reconstructed. Inferred phylogenies were assessed for their agreement with the commonly accepted family tree of yeast species (see figure 1; we found excellent agreement among the inferred trees assessed using the *clann *software package [38]) and the widely accepted phylogenies for the yeast and nematode species, which are shown in figure 1. For further analyses we chose to use the maximum likelihood rate.

#### Statistical analysis

In order to be able to compare evolutionary rates for as many proteins as possible we defined the averaged relative evolutionary rate of each protein *i *via



where *v*_*i *_is the number of comparisons from which an evolutionary rate can be estimated.

We generally found that analysis of the dependence of *R *= {*R*_1_, *R*_2_, ..*R*_*n*_} on the number of interactions *etc. *behaved similarly to analysis of the species specific rates. We used ANOVA [25] and partial correlation coefficients to study the impact the different factors had on the evolutionary rates. All analyses were performed using the R statistical environment and the NetZ package (available from the authors). In order to investigate the relative influence of the various factors (number of interactions, expression levels, GO-data [20]) we used linear and generalized linear regression modelling (implemented in R). The Akaike information criterion (AIC) [16,21] was used to distinguish among the different nested submodels of the full model. The model which has the smallest AIC (defined as 2(-1 k(*θ*) + 2*v*) where 1 k(*θ*) is the log-likelihood of a – potentially vector-valued – parameter *θ*, and *v *is the number of parameters) is the model which offers the best (in an information sense [21]) description of the data. The full model included the number of interactions, expression levels (or CAI in the case of *C. elegans*) and GO-data as explanatory variables. When comparing evolutionary rates of interacting proteins the evolutionary rate at the interacting protein was added as an explanatory variable. The AIC (and related approaches [21]) aims to identify a statistical model that offers the most efficient description of the data (in an information theoretic sense) from a set of trial models.

**Figure 7:**
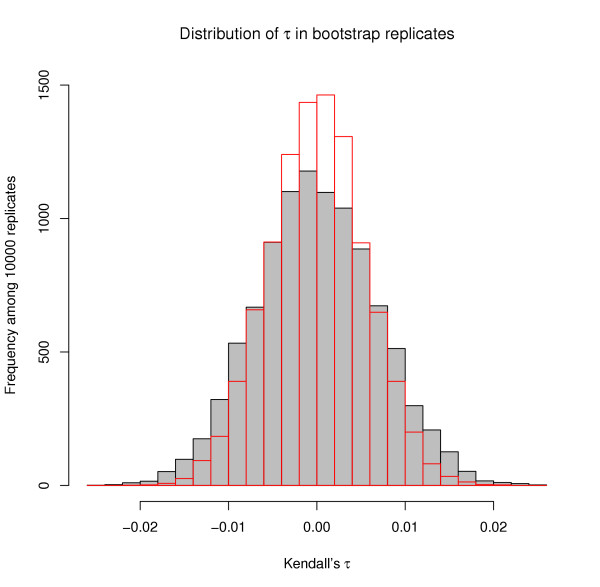
**Confidence intervals calculated with and without including the network structure**. Distribution of Kendall's *τ *measuring the expected correlation between the expression levels of interacting proteins in *S. cerevisiae*. The grey distribution has been calculated under the correct empirical Null distribution, where pairs of interacting proteins are chosen such that the degree distribution of the re-sampled protein network is the same as that of the true network. The red bars indicate the distribution obtained under the conventional (and inadequate) Null model where pairs of proteins are chosen entirely at random. Including the network structure into the bootstrap procedure leads to a broader distribution.

We explicitly incorporated the network structure into the statistical analysis. This is necessary if there is reason to believe that properties of the network may determine aspects of the evolutionary history, for example when we want to test if the evolutionary rates of interacting proteins are correlated. Here we use resampling or bootstrap procedures [18] to determine if properties (*e.g. *expression levels, evolutionary rates, connectivities) of interacting proteins are more similar than would be expected to occur by chance. If instead we had paired proteins completely at random we would potentially have masked confounding effects due to the network (for example if expression depends strongly on a protein's network properties, *i.e. *connectivity). In our network-aware resampling procedure we therefore pick each protein with a probability that is proportional to its number of interaction partners. Each bootstrap replicate is thus also a sample with the correct nodal properties and (statistically) the same degree distribution as the true network. In the structural analysis of networks the need to account for network properties in the construction of the correct Null model has long been realized [14,17] but this is, to our knowledge, the first time that such a topologically correct Null model has been applied to the evolutionary analysis of network data. As an illustration figure 7 shows the bootstrap distribution of correlation coefficients of expression levels in yeast for the correct Null model and for the model where proteins are paired completely at random. Ignoring the correlation of the data introduced by the underlying network structure reduces the bootstrap confidence intervals considerably (we find that the two-sided 95% CIs are reduced by approximately 20% compared to the network aware bootstrap replicate). This mirrors the effects observed in population and evolutionary genetics where the underlying genealogy/phylogeny increases the CIs compared to the case of truly independent observations.

Routines used to perform the statistical analysis of the network data are collected in the NetZ package which can be obtained from the corresponding author.

## Authors' contributions

IA collected the data, performed the phylogenetic analysis, helped with the statistical analysis and with writing the manuscript. JS calculated the codon adaptation index, helped with the evolutionary and statistical analysis and writing the paper. JA, DH and SB helped with data retrieval, the evolutionary analysis of the PIN data, and writing the manuscript. MPHS devised the study, performed the statistical analysis and wrote the manuscript.

## Supplementary Material

Additional File 1 We have used GO classifications [20] to evaluate the extent to which function (table S1), biological process (table S2), or celluar compartment (table S3) of a protein may influence the evolutionary rate of proteins in *S. cerevisiae*. Click here for file
