# Supplementary material for: Comparative analysis of the Saccharomyces cerevisiae and Caenorhabditis elegans protein interaction networks
Source: BMC Evol Biol. 2005 Mar 18;5:23. doi: 10.1186/1471-2148-5-23 (PMC1079807; doi:10.1186/1471-2148-5-23)
Supplement: Additional File 1 — We have used GO classifications [20] to evaluate the extent to which function (table S1), biological process (table S2), or celluar compartment (table S3) of a protein may influence the evolutionary rate of proteins in S. cerevisiae. [file 1471-2148-5-23-S1.pdf]

Table S1 - Estimated evolutionary rates for proteins with different functions

Averaged estimated evolutionary rates for proteins belonging to each functional class in the gene ontology.

Function	Number	R2	M2	B2	C2	K2	A2	P2
no GO data	560	0.73	0.14	0.15	0.41	0.43	0.57	0.59
chaperone	72	0.55	0.07	0.11	0.31	0.36	0.46	0.56
DNA-binding	121	0.68	0.13	0.15	0.40	0.46	0.60	0.59
enzyme-regulation	110	0.66	0.12	0.14	0.41	0.40	0.61	0.61
helicase	48	0.63	0.09	0.12	0.33	0.33	0.47	0.53
hydrolase	278	0.61	0.12	0.13	0.34	0.37	0.53	0.59
isomerase	26	0.44	0.07	0.11	0.26	0.27	0.42	0.46
ligase	74	0.56	0.12	0.10	0.28	0.28	0.45	0.50
lyase	63	0.50	0.06	0.10	0.28	0.27	0.39	0.49
function unknown	1484	0.72	0.17	0.19	0.47	0.48	0.63	0.68
motor activity	16	0.66	0.13	0.18	0.51	0.49	0.48	0.53
nucleotidyltransferase	63	0.57	0.06	0.10	0.28	0.30	0.46	0.54
oxidoreductase	158	0.45	0.07	0.10	0.29	0.31	0.43	0.49
peptidase	90	0.56	0.08	0.11	0.30	0.31	0.49	0.50
phosphoprotein-phosphatase	46	0.54	0.08	0.12	0.35	0.37	0.51	0.54
protein-binding	216	0.70	0.13	0.17	0.41	0.45	0.61	0.62
protein-kinase	107	0.61	0.11	0.14	0.38	0.39	0.55	0.63
RNA-binding	178	0.68	0.12	0.15	0.37	0.38	0.58	0.60
signal-transducer	56	0.65	0.08	0.14	0.34	0.36	0.59	0.62
structural-molecule	211	0.62	0.09	0.12	0.35	0.33	0.52	0.58
transcription-regulator	242	0.73	0.13	0.16	0.44	0.44	0.63	0.68
transferase	273	0.57	0.11	0.12	0.32	0.37	0.50	0.56
translation-regulator	44	0.53	0.06	0.09	0.29	0.28	0.45	0.49
transporter	237	0.57	0.11	0.11	0.33	0.35	0.49	0.57

Table S2 - Estimated evolutionary rates for proteins involved in different biological processes

Averaged estimated evolutionary rates for proteins which have been assigned to different biological processes in the gene ontology.

Process	Number	R2	M2	B2	C2	K2	A2	P2
no Go data	621	0.68	0.14	0.14	0.38	0.37	0.54	0.57
amino-acid and derivative metabolism	95	0.48	0.07	0.09	0.23	0.28	0.41	0.48
process unknown	1003	0.69	0.17	0.19	0.47	0.48	0.61	0.67
budding	24	0.72	0.07	0.18	0.36	0.50	0.55	0.54
carbohydrate-metabolism	89	0.43	0.06	0.10	0.29	0.35	0.42	0.45
cell-cycle	155	0.72	0.15	0.16	0.47	0.51	0.65	0.69
cell homeostasis	33	0.63	0.12	0.11	0.35	0.41	0.55	0.57
cellular respiration	41	0.59	0.05	0.13	0.36	0.39	0.54	0.56
cell-wall organization and biogenesis	87	0.57	0.15	0.15	0.36	0.41	0.56	0.55
coenzyme and prosthetic group metabolism	46	0.55	0.08	0.10	0.32	0.33	0.46	0.55
conjugation	42	0.74	0.17	0.21	0.50	0.47	0.66	0.74
cytokinesis	51	0.71	0.15	0.14	0.38	0.39	0.59	0.66
cytoskeleton organization and biogenesis	74	0.75	0.12	0.18	0.41	0.36	0.56	0.56
DNA-metabolism	204	0.71	0.11	0.17	0.39	0.43	0.57	0.61
electron-transport	6	0.35	0.04	0.06	0.23	0.19	0.36	0.56
energy-pathways	19	0.35	0.09	0.12	0.23	0.35	0.45	0.47
lipid metabolism	96	0.58	0.08	0.11	0.33	0.34	0.52	0.58
meiosis	67	0.70	0.22	0.19	0.47	0.48	0.59	0.64
membrane-organization and biogenesis	14	0.63	0.11	0.14	0.33	0.43	0.52	0.61
morphogenesis	18	0.70	0.11	0.20	0.45	0.47	0.65	0.69
nuclear-organization and biogenesis	66	0.72	0.12	0.18	0.46	0.46	0.66	0.71
organelle-organization and biogenesis	74	0.70	0.13	0.15	0.47	0.43	0.61	0.66
protein-biosynthesis	225	0.53	0.08	0.10	0.29	0.31	0.45	0.51
protein-catabolism	88	0.58	0.12	0.12	0.34	0.32	0.51	0.54
protein-modification	214	0.69	0.13	0.14	0.39	0.42	0.57	0.62
pseudohyphal growth	38	0.64	0.15	0.17	0.40	0.43	0.57	0.60
response to stress	139	0.62	0.11	0.13	0.35	0.39	0.54	0.59
ribosome-biogenesis and assembly	46	0.57	0.08	0.11	0.26	0.29	0.44	0.50
RNA-metabolism	274	0.68	0.13	0.15	0.36	0.38	0.55	0.60
signal-transduction	47	0.68	0.13	0.17	0.43	0.42	0.61	0.63
sporulation	32	0.59	0.15	0.19	0.53	0.48	0.62	0.74
transcription	234	0.70	0.11	0.15	0.40	0.41	0.60	0.62
transport	372	0.65	0.12	0.14	0.38	0.39	0.56	0.61
vesicle-mediated transport	112	0.63	0.14	0.12	0.37	0.40	0.60	0.65
vitamin-metabolism	27	0.47	0.07	0.11	0.33	0.31	0.45	0.57

Table S3 - Estimated evolutionary rates for proteins in different cellular compartments
Averaged estimated evolutionary rates for proteins with different cellular compartment assignments in the gene-ontology. The is comparatively little variation between compartments and different species comparisons provide qualitatively similar results.

Process	Number	R2	M2	B2	C2	K2	A2	P2
no Go data	556	0.72	0.15	0.16	0.40	0.41	0.58	0.59
bud	64	0.73	0.17	0.19	0.44	0.49	0.64	0.67
cell-cortex	18	0.84	0.10	0.15	0.44	0.50	0.68	0.74
cellular-component-unknown	478	0.70	0.18	0.22	0.50	0.50	0.63	0.67
cell-wall	51	0.55	0.14	0.19	0.38	0.42	0.56	0.62
chromosome	31	0.71	0.08	0.14	0.39	0.43	0.57	0.62
cytoplasm	841	0.60	0.12	0.13	0.35	0.36	0.52	0.56
cytoplasmic-vesicle	30	0.59	0.15	0.14	0.37	0.44	0.60	0.67
cytoskeleton	60	0.67	0.11	0.15	0.41	0.41	0.62	0.59
endo-membrane system	60	0.81	0.16	0.19	0.47	0.43	0.66	0.71
endoplasmic-reticulum	192	0.65	0.11	0.13	0.37	0.42	0.56	0.61
extracellular	10	0.59	0.09	0.13	0.37	0.45	0.53	0.65
Golgi-apparatus	55	0.71	0.19	0.14	0.38	0.41	0.62	0.67
membrane	168	0.67	0.15	0.16	0.42	0.40	0.57	0.64
membrane-fraction	42	0.72	0.17	0.18	0.42	0.40	0.60	0.68
microtubule-organizing-center	38	0.80	0.14	0.19	0.57	0.52	0.69	0.66
mitochondrial-membrane	105	0.58	0.09	0.10	0.36	0.36	0.51	0.57
mitochondrion	248	0.62	0.10	0.14	0.38	0.39	0.53	0.58
nucleolus	115	0.62	0.10	0.13	0.33	0.32	0.47	0.56
nucleus	1296	0.67	0.13	0.15	0.40	0.42	0.57	0.61
peroxisome	22	0.60	0.11	0.17	0.37	0.44	0.55	0.59
plasma-membrane	125	0.60	0.14	0.15	0.36	0.40	0.55	0.62
ribosome	118	0.55	0.08	0.10	0.30	0.31	0.49	0.54
site of polarized growth	5	0.63	0.10	0.21	0.39	0.32	0.59	0.82
vacuole	45	0.67	0.18	0.18	0.41	0.43	0.56	0.64
